# Fluid Micro-Reservoirs Array Design with Auto-Pressure Regulation for High-Speed 3D Printers

**DOI:** 10.3390/mi7110202

**Published:** 2016-11-07

**Authors:** Moshe Einat

**Affiliations:** Faculty of Engineering, Ariel University, Ariel 40700, Israel; einatm@ariel.ac.il; Tel.: +972-3-9066-388

**Keywords:** high-speed three dimensional (3D) printers, reservoirs, pressure regulation

## Abstract

Three dimensional (3D) printing technology is rapidly evolving such that printing speed is now a crucial factor in technological developments and future applications. For printing heads based on the inkjet concept, the number of nozzles on the print head is a limiting factor of printing speed. This paper offers a method to practically increase the number of nozzles unlimitedly, and thus to dramatically ramp up printing speed. Fluid reservoirs are used in inkjet print heads to supply fluid through a manifold to the jetting chambers. The pressure in the reservoir’s outlet is important and influences device performance. Many efforts have been made to regulate pressure inside the fluid reservoirs so as to obtain a constant pressure in the chambers. When the number of nozzles is increased too much, the regulation of uniform pressure among all the nozzles becomes too complicated. In this paper, a different approach is taken. The reservoir is divided into an array of many micro-reservoirs. Each micro-reservoir supports one or a few chambers, and has a unique structure with auto-pressure regulation, where the outlet pressure is independent of the fluid level. The regulation is based on auto-compensation of the gravity force and a capillary force having the same dependence on the fluid level; this feature is obtained by adding a wedge in the reservoir with a unique shape. When the fluid level drops, the gravitational force and the capillary force decrease with it, but at similar rates. Terms for the force balance are derived and, consequently, a constant pressure in the fluid micro-reservoir segment is obtained automatically, with each segment being autonomous. This micro reservoir array is suggested for the enlargement of an inkjet print head and the achievement of high-speed 3D printing.

## 1. Introduction

Inkjet technology has made continuous developments since the 1950s [1]. In 1977, the emergence of the drop on demand (DOD) concept [2] quickly paved the way for affordable home printers. Printing has now moved beyond ink on paper, entering a truly revolutionary technological phase [3]. For instance, new avenues of printing include printing antennas with conductive ink [4] as well as printing phased arrays and polymer printing [5]. Other innovations under exploration are printing of semiconductors and biological applications [6]. The same technology is also used to create micro pumps that drive fluid in micro systems. Certain three dimensional (3D) printers are also based on inkjet technology, and nowadays 3D printing may become a revolutionary technology in many disciplines. For example, thermal inkjet printers were used to print viable mammalian cells [7] and human microvasculature tissues [8]. Recently, a 3D printed circuit board (PCB) printer that may revolutionize the electronic development process was developed [9]. This printer has already printed multilayer PCB boards with blind via a USB connector, and it has the possibility to print miniature waveguides and antennas integrated to the PCB. It seems that, today, inkjet technology is penetrating into unexpected and unprecedented regimes in which the unique qualities of the inkjet can be used to perform new tasks with just a few drops of various fluids, a mere pico-liters volume.

A general limiting factor for the entire 3D printing technologies is the printing speed. Although there are different concepts of 3D printing, model printing still takes too much time for many applications. A breakthrough to obtain a high-speed printer that can form a printed model on the scale of seconds is needed.

The DOD technology is generally divided into thermal inkjet (where the ejection is done by micro-boiling [10] generated with a micro heater) and piezo inkjet [11] (where the ejection is done by a piezo actuator). In both concepts, there is a small chamber filled with fluid, and when the print head is in position, an electrical pulse is sent to the heater or actuator. As a result, a local and temporal up-rise of the pressure is created in the chamber. This up-rise pushes the fluid towards a small hole in the chamber—the nozzle. A drop is then ejected to the media and printing is obtained [12]. After the pulse, the pressure in the chamber returns to its former value and the chamber is ready for the next drop.

In order to obtain uniform drops, there is a general demand for keeping the relevant parameters unchanged during the operation of the print head. One important parameter is the fluid pressure in the chamber. The initial pressure in the chamber is a participating factor in determining ejected drop characteristics. Therefore, it is imperative that chamber pressure remains unchanged during this time. Recall that the chamber is connected to a reservoir filled with the fluid; thus, a change in reservoir fluid level may influence the pressure in the chamber. This problem was recognized in the early days of inkjet technology development and even at that time solutions had been proposed. In a publication of IBM from 1973 [13], a complicated reservoir is described in which the pressure is kept by regulating the air and ink penetration into the reservoir from a supply bottle. Later, the problem had been addressed extensively [14,15,16,17,18], with various solutions developed and patented to regulate the pressure in the reservoir, consequently keeping the chamber pressure unchanged during the decrease in ink level in the print head. Such solutions included the use of pistons, membranes, springs, variable volume structure in the ink reservoir for expanding as ink is drawn out, and many more concepts.

Clearly, this problem is fundamental to inkjet technology. Moreover, when the number of nozzles connected to the reservoir is increased, the connecting manifold becomes more complex, making the preservation of regulated pressure in all the nozzles the limiting factor of print head enlargement. Therefore, for more than a few thousand nozzles, other solutions are needed, such as cascading several heads. This is actually a major limiting factor towards the enlargement of print heads and thus printing speed [19,20].

In this paper, an elegant and rather simple solution to the problem of pressure regulation in the reservoir is suggested. The single reservoir is divided into an array of many micro-reservoirs. The entire head is segmented. Each segment includes a micro reservoir and a nozzle (or a few nozzles). The micro reservoir has a unique structure allowing a constant pressure at its base regardless of the fluid level it contains. Therefore, no external means for pressure regulation are needed and the surrounding system can be simplified. Moreover, adding segments is practically unlimited. The print head can be sized as a large liquid crystal display (LCD) screen, having nozzles numbered similar to those of light-emitting diodes (LEDs) on the screen. Therefore, printing speed can be dramatically increased. With such a large print head, objects of significant size can be printed in seconds instead of hours, as current technology allows. The arrangement of such a 2D print head is described in Reference [19], where the ink delivery system (with regular reservoirs) is outlined and tested. Experimental arrangement of the nozzle chambers of the print head, as well as experimental printing results, are reported in Reference [20]. In this paper the unique structure of the micro-reservoir allowing constant pressure to the ink delivery system is reported. Combining the above elements results in an immediate printing of an entire layer and thus high-speed 3D printing.

## 2. Reservoir Structure and Concept

**Figure 1:**
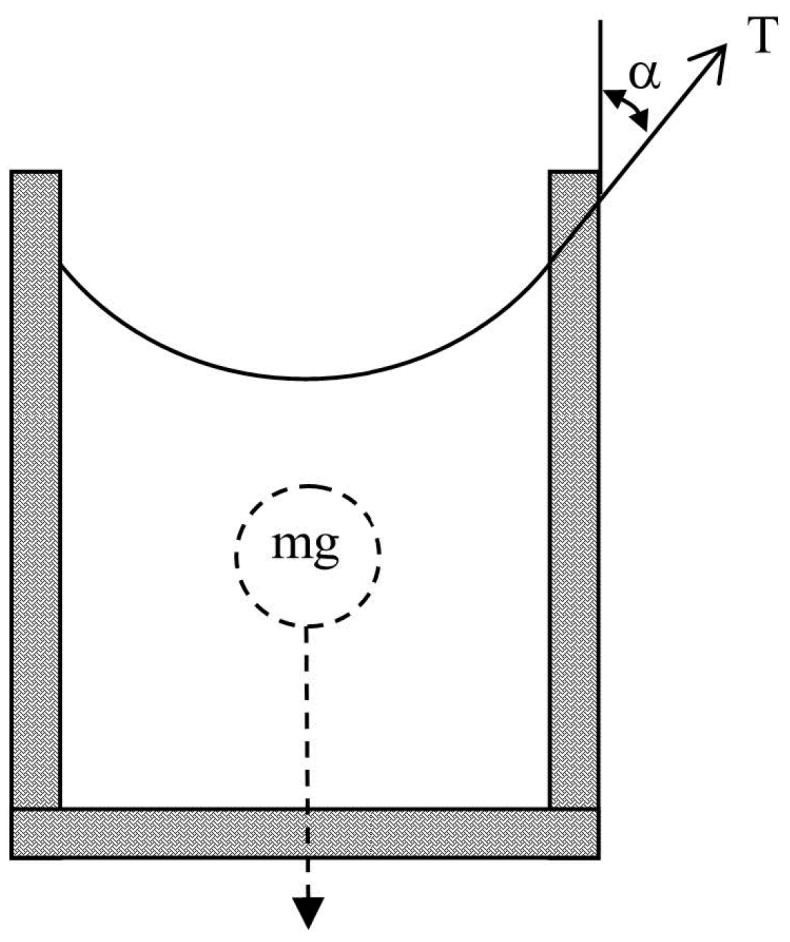
A fluid reservoir filled up to a certain level, the base pressure is determined by the gravitational and capillary forces, and depends on the fluid level.

Let us first assume a regular fluid reservoir filled up to a certain level (Figure 1). The pressure at the base of the reservoir is a sum of two forces divided by the area of the base. The forces are the weight of the fluid pushing downwards, and the capillary force at the contact line of the fluid and the reservoir pulling upwards.

In large reservoirs, the capillary force is much smaller and can safely be neglected. However, as the reservoir becomes smaller (or porous) this force becomes relatively stronger and can even be the dominant one. In the following derivation, the capillary force will be taken into account. The total force operating on the fluid is [21]:

*F* = *mg* − *T*_UP_ (1)

where *m* is the fluid mass, *g* is the gravitational constant and *T*_UP_ is the capillary force portion directed upwards, and is calculated as:

*T*_UP_ = *T*cosα = σ*L*cosα (2)

*T* is the total capillary force and is calculated as the surface tension σ multiplied by the contact line length between the fluid and the chamber *L*. The direction of *T* is along the contact angle α. Taking into account the chamber cross-section *S*, the specific gravity of the fluid ρ and fluid level *h*, Equation (1) becomes:

*F*(*h*) = ρ*Vg − T*_UP_ = ρ*Sgh +* σ*L*cosα (3)

where *V* is the fluid volume. As seen, the force is dependent on the fluid level *h*. Therefore, the pressure in the chamber base *P* is also dependent on the fluid level:

*P*(*h*) = *F*(*h*)/*S* = ρ*gh +* σ*L*cosα/*S* (4)

As seen in Equation (4), the pressure decreases together with the fluid level. In light of the above, a critical question forms: is there a possibility to develop a structure where the pressure is independent of the fluid level?

Such a structure, if possible, would clearly have superior properties regarding inkjet and micro-pump applications.

Looking at Equation (4), if the structure of the reservoir could be delicately chosen to obtain a unique and desired influence on the magnitude of the capillary force, it may be possible to compensate the change in the gravitational force. Since the gravitational force on the fluid decreases as the fluid level drops, a reservoir structure might be found in which a similar dependence of the capillary force on the fluid level occurs. This, in turn, means that the capillary force also drops at the same rate as the gravitational force. Thus, the changes in the two forces compensate each other and the level change does not influence the pressure at the base. Such a solution is presented in the next paragraph.

## 3. The Reservoir Model

**Figure 2:**
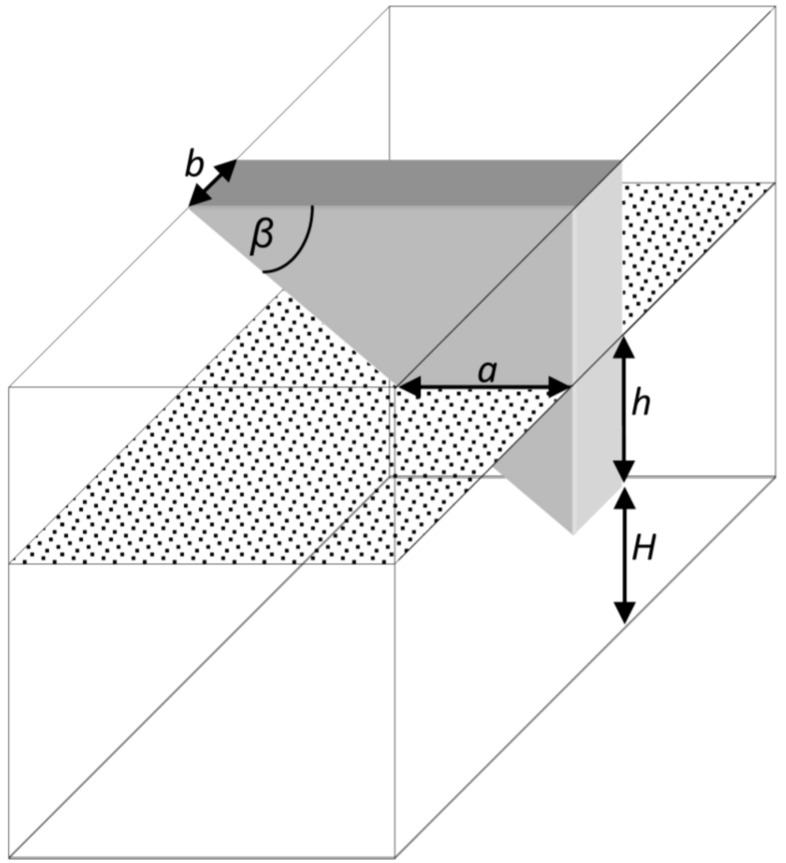
The auto-pressure regulation reservoir. A wedge is inserted to add compensating capillary force, performing balance with the gravitation force. Constant pressure is obtained at the base regardless of the fluid level *h*.

Let us insert a wedge into the chamber as seen in Figure 2. The height is *H* where the wedge begins, and *h* is the fluid level above this height. The wedge width is *b*, and β is the angle of the wedge. The fluid level determines *a* which is the width of the wedge that is covered with fluid.

Repeating the derivation for this chamber, the volume of the fluid is almost the same, calculated for a cube structure, subtracting the wedge volume:

(5) $$V(h)=S\cdot H+S\cdot h-\frac{1}{2}ab\cdot h=S\cdot H+S\cdot h-\frac{b}{2\tan\beta }\cdot h^{2}$$

where *S* is the base area and *H*, *h*, *a*, *b*, and β are as noted in Figure 2. When *b* is kept small enough, the volume (and gravity) does not change much due to the wedge, but the capillary force is strongly affected. Calculating the capillary force, we get three components:

(6) $$T_{\mathrm{UP}}(h)=\sigma (L-b)\cos\alpha +\sigma b\cos\alpha_{b}+\frac{2\sigma \cos\alpha }{\tan\beta }\cdot h$$

where α*_b_* is the contact angle in the wedge slope (here *L* is the contact line length between the fluid and the chamber without the wedge). As can be seen, the first component is due to the reservoir walls and is similar to the case of a standard reservoir. The second one is negligible when *b* is small enough and does not contribute much. The last component is the new important part. As seen, this component is dependent on *h* and can potentially compensate for the dependence of the gravitational force on *h*. If all the components contributing to the base pressure in the modified chamber are collected and organized according to the dependence on *h*, the pressure is obtained as follows:

*P*(*h*) = ρ*Vg − T*_UP_ = *K* + *K*_1_·*h + K*_2_·*h*^2^ (7)

where *K* is a constant containing all the components that are not dependent on *h* (both from gravity as well as the capillary force):

*K* = ρ*gH −* [σ(*L− b*)cosα *−* σ*b*cosα*_b_*]/*S* (8)

*K*_1_ is the coefficient of *h*:

(9) $$K_{1}=\left[\rho g-\frac{2\sigma \cos\alpha }{S\tan\beta }\right]$$

and *K_2_* is the coefficient of *h*^2^:

(10) $$K_{2}=-\frac{\rho gb}{2S\tan\beta }$$

The last term in Equation (7) contains a dependence on *h*^2^, but it is also dependent on *b*, which is the wedge width. If we enforce a small *b*, as small as the limit of practical production in microelectromechanical systems (MEMS) technology, we can neglect this term.

The second term in Equation (7) contains a dependence on *h*, which still varies as the fluid level drops. However, with proper choice of the constants in this term, it is possible to obtain a zero coefficient meaning: *K*_1_ = 0. The condition for that will be:

(11) $$\rho g=\frac{2\sigma \cos\alpha }{S\tan\beta }$$

As can be seen, if the above conditions are fulfilled, the final expression for the pressure at the base of the modified chamber *P* becomes:

*P* = *K* = ρ*gH +* [σ(*L− b*)cosα *+* σ*b*cosα*_b_*]/*S* (12)

making it independent of *h*. Therefore, a chamber with a constant pressure regardless of its fluid level is obtained.

For example, assuming a squared modified chamber filled with water has the following parameters:

σ = 0.072 N/m
ρ = 997 kg/m^3^
*L* = 2.5 mm
*S* = 6.25 mm^2^
*g* = 9.81 m/s^2^
α = 24.45°
β = 65°

The two terms in *K*_1_ (Equation (9)) practically cancel each other out:

(13) $$K_{1}=\left[\rho g-\frac{2\sigma \cos\alpha }{S\tan\beta }\right]=9780-9780\;\sim \;0{\;(N/m}^{3})$$

And cancellation of the dependence on the fluid level is obtained.

Also, determining *K* can be done in order to choose the constant pressure; positive as well as negative values are possible with proper determination of the constants.

The calculation is repeated for other values of surface tension typical for inks and the results are detailed in Table 1. The rest of the parameters are kept as above.

**Figure 3:**
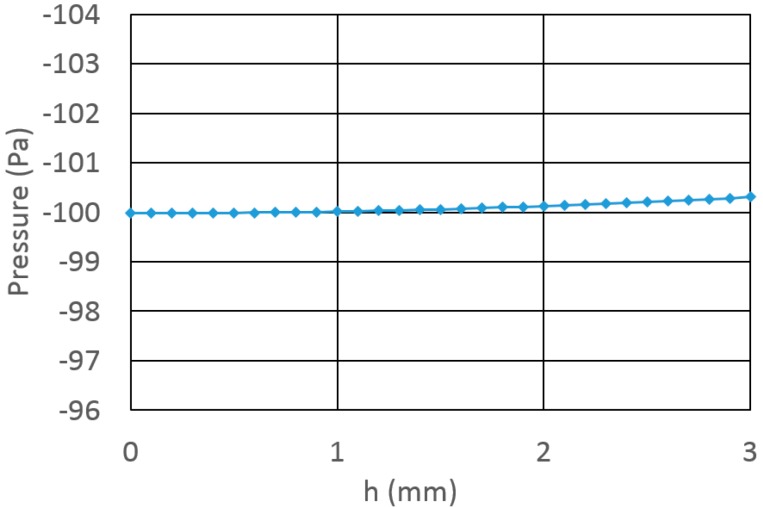
The pressure dependence on fluid level *h*. Negligible variation in the pressure (~0.3%) is predicted.

In order to obtain the predicted stability of the pressure as the level drops, a calculation was done according to Equation (7) (without any negations). As a basis pressure, a value of −100 Pa was taken. The wedge width b is taken to be 100 μm in this calculation. The results are shown in Figure 3. As seen, even with a span of 3 mm in the fluid level, the pressure shifted in less than 0.4 Pa.

## 4. Conclusions

In this paper, an inkjet micro-reservoir with inherent structure-based pressure regulation is presented. The structure-based regulation is obtained by inserting a wedge that adds a capillary force which is dependent on the fluid level. This dependent capillary force is equal in magnitude to, and opposite in direction from, the gravitational force at any fluid level. Therefore, a base pressure that is independent of the fluid level is obtained. An example is given to demonstrate the concept.

An important feature of this solution is related to a case where a plurality of reservoirs is in use. In such a case, with standard reservoirs, the pressure within will not be uniform among the reservoirs. However, with the suggested reservoir, even when the level of the fluid is not uniform in the reservoirs, the pressure would still be uniform and constant—an important solution for a segmented system. This reservoir can be built with MEMS technology to obtain a large area of similar reservoirs. A huge array can be built where each reservoir is autonomous and independent of its surrounding reservoirs. As a result, this reservoir enables the enlargement of a print head to include an enormous number of nozzles and thus achieve a high-speed 3D print head where an entire layer is printed simultaneously without scanning.

An example for such a system (with standard reservoirs) is presented in References [19,20], where a two-dimensional print head is presented. In such a system, matrices of micro-reservoirs are needed, but the fluid level would not be equal among them. However, if a reservoir as presented in this paper would be used, a constant pressure could be achieved in all reservoirs.

Along with the suggested design, several aspects should be addressed. As seen, the pressure is greatly dependent on the geometry. How will imperfections influence the performance? Clearly, the answer is dependent on the technology used to form the reservoirs. However, from the graph in Figure 3 it is seen that even though a rather thick wedge had been considered (100 μm), good results are still predicted. The expected imperfections should be much smaller and therefore it seems that there is a wide range of practical parameters with which the reservoir can be realized. Better technology (resolution, accuracy) will allow better results.

Another aspect is the influence of dust and pollution that may interfere and clog the system. This is a well-known problem in inkjet systems and many means have been used to attempt pollution removal. Usually this task is difficult because the system is closed and the only way out is through the nozzle which is rather small. Clearly, such pollution can interfere in unexpected ways. However, in the suggested reservoir there is a unique remedy for this problem. This reservoir can be reached from the upper side, which is wide. Therefore, a back-priming can be done; i.e., sucking out all the fluid from the wide side (with the pollution) and replenishing a new clean fluid.

A unique property of the presented micro reservoir is its quasi-static operation. Since the reservoir feeds only one (or a few) nozzles, replenishing can be done rarely and most of this operation is done in a static manner, where no fluid is added. As a result, dynamic effects can be minor and negligible. It should be noted that cross talk between neighboring nozzles is also a dynamic phenomenon, which would still be present if the proposed reservoir would support multiple nozzles. Nevertheless, the problem is significantly reduced for a low number of nozzles connected to the same reservoir, and totally eliminated when every nozzle has its own reservoir.

Another limitation related to a high speed inkjet 3D printer is the ultraviolet (UV) curing which is done after each layer in most inkjet 3D printers. This process might be the next bottle neck towards high speed printing. A solution for this issue may come from chemical research and development of advanced fluids with a fast curing mechanism that can be integrated to a fast inkjet print head.

The same solution is applicable for micro-pumps and other MEMS devices utilizing fluids where constant pressure is required. It should be noted that with proper tuning of the parameters, both positive and negative constant pressure could be obtained for various applications.

## Figures and Tables

**Table 1:** Results for different surface tension values.

σ	α	β
0.072 N/m	24.45°	65°
0.04 N/m	28.48°	49°
0.025 N/m	34.45°	34°
